# Sustained Delivery of Bioactive GDNF from Collagen and Alginate-Based Cell-Encapsulating Gel Promoted Photoreceptor Survival in an Inherited Retinal Degeneration Model

**DOI:** 10.1371/journal.pone.0159342

**Published:** 2016-07-21

**Authors:** Francisca S. Y. Wong, Calvin C. H. Wong, Barbara P. Chan, Amy C. Y. Lo

**Affiliations:** 1 Department of Ophthalmology, Li Ka Shing Faculty of Medicine, The University of Hong Kong, Hong Kong, China; 2 Tissue Engineering Laboratory, Department of Mechanical Engineering, Faculty of Engineering, The University of Hong Kong, Hong Kong, China; 3 Research Centre of Heart, Brain, Hormone and Healthy Aging, Li Ka Shing Faculty of Medicine, The University of Hong Kong, Hong Kong, China; Purdue University, UNITED STATES

## Abstract

Encapsulated-cell therapy (ECT) is an attractive approach for continuously delivering freshly synthesized therapeutics to treat sight-threatening posterior eye diseases, circumventing repeated invasive intravitreal injections and improving local drug availability clinically. Composite collagen-alginate (CAC) scaffold contains an interpenetrating network that integrates the physical and biological merits of its constituents, including biocompatibility, mild gelling properties and availability. However, CAC ECT properties and performance in the eye are not well-understood. Previously, we reported a cultured 3D CAC system that supported the growth of GDNF-secreting HEK293 cells with sustainable GDNF delivery. Here, the system was further developed into an intravitreally injectable gel with 1x10^4^ or 2x10^5^ cells encapsulated in 2mg/ml type I collagen and 1% alginate. Gels with lower alginate concentration yielded higher initial cell viability but faster spheroid formation while increasing initial cell density encouraged cell growth. Continuous GDNF delivery was detected in culture and in healthy rat eyes for at least 14 days. The gels were well-tolerated with no host tissue attachment and contained living cell colonies. Most importantly, gel-implanted in dystrophic Royal College of Surgeons rat eyes for 28 days retained photoreceptors while those containing higher initial cell number yielded better photoreceptor survival. CAC ECT gels offers flexible system design and is a potential treatment option for posterior eye diseases.

## Introduction

It is estimated that 285 million people are visually impaired or blind around the world [1]. Limited treatment options are yet available for common sight-threatening diseases such as degenerative retinopathies, diabetic retinopathies, glaucoma, cytomegalovirus (CMV) retinitis, uveitis, and retinal vein and artery occlusions due to the lack of effective drug delivery system [2]. Although repeated administrations of therapeutics through the invasive intravitreal route are clinically performed to improve local drug availability, this type of drug administration is plagued by heavy treatment burden on physicians and patients as well as cumulative risks and potential complications such as infectious endophthalmitis, elevated intraocular pressure, retinal vascular occlusion and rhegmatogenous retinal detachment [3]. Effective sustainable drug delivery platforms are warranted.

Encapsulated-cell therapy (ECT) is an attractive approach for delivering freshly synthesized therapeutics targeting a wide range of vision-threatening diseases in the posterior eye [4]. Since the pioneering work by Chang et al., ECT has been evolved into a new area of biomedical research and applied to a plethora of diseases and targeted locations [5]. By encapsulating and immunoisolating drug-secreting cells of autogeneic, allogeneic and xenogeneic sources in a semipermeable membrane and/or matrix, neuroactive agents can be continuously delivered at the target site. Since ECT does not genetically alter the host cells and can overcome the need of regular replacement of exhausted reservoir-type implants, disturbance to the host system can potentially be minimized. Hydrogel materials are often applied as an encapsulating matrix alone or as an ECT device matrix-filler to improve cell viability and avoid aggregation of diving cells inside the semi-permeable membrane. Their permeability can be tailored to fit the metabolic requirements of the encapsulated cells so as to support their prolonged survival and functioning. Naturally-occurring hydrogel biomaterials such as alginate and collagen are widely studied since they have good biocompatibility, low immunogenicity, gentle gelation mechanism that can take place in the presence of cells, and are economical and readily available [6]. Collagen constitutes the greatest quantity of the total proteins in the human body, and is a major component of the extracellular matrix (ECM). It is also one of the most extensively used ECM materials in neural tissue engineering. Alginate is a linear anionic polysaccharide commonly purified from brown algae and is the most studied biomaterial for ECT. As alginate is biologically inert, some studies have functionalized it with ECM or ECM-mimicking components to improve cell survival rate through enhancing cell-material adhesion and interactions [7–10]. ECT matrix composed of a collagen-alginate (CAC) interpenetrating network (IPN) integrates the physical and biological strength of its constitutional biomaterials. IPN is formed when at least one polymer is cross-linked within the immediate presence of the other, without any covalent bonds between them and cannot be separated unless chemical bonds are broken. CAC hydrogel demonstrated better mechanical properties *in vitro* when compared with alginate or collagen scaffolds alone [11–13]. However, parameters essential for tuning the properties of CAC-based ECT systems as well as their drug delivery performance *in vivo* are not well-understood.

GDNF is a member of the TGF-β superfamily and is a potent neuroprotective agent that acts on neuronal cells in both central nervous system (CNS) and peripheral nervous systems at different stages of development [14–16]. GDNF has also been applied in preclinical and clinical trials to treat CNS degeneration and has attracted attention in ameliorating retina degenerations [17–20]. GDNF has shown to promote photoreceptor and ganglion cell survival when applied *in vitro* and in a range of animal models [20]. However, as GDNF has a relatively short half-life [21], sustained delivery is warranted to overcome the need for repeated intravitreal injections. Some delivery systems investigated for prolonged GDNF delivery include polymer-release systems [22–25], cellular/cell replacement therapy [26–28], and viral or non-viral-mediated gene therapy [29–34]. No attempt has been made to date to deliver GDNF via ECT for ocular applications to our knowledge.

Previously, we have reported a 3D collagen microencapsulation culture system that supported the growth of GDNF-secreting HEK293 cells, enhanced their protein secretion rate, and achieved sustained GDNF secretion for up to 30 days *in vitro* [35]. Inclusion of alginate in the collagen scaffold improved its cell immobilization power, supported cell growth, and controlled the pattern of GDNF secretion [9]. In this study, the CAC ECT platform was further developed into an intravitreally injectable gel for treating posterior eye disorders in a well-studied animal model of recessive retinal pigmentosa (RP), the dystrophic Royal College of Surgeons (RCS) rat. These rats have a mutation in the tyrosine kinase *Mertk* gene that is mainly expressed in retinal pigment epithelial (RPE) cells, which prevents them from phagocytizing shed photoreceptor outer segment (OS) debris accumulated in the subretinal space, leading to photoreceptor cell loss [36–39]. In this study, the factors impacting encapsulated cell growth, including alginate concentration and initial cell density, were firstly investigated. Potentials of sustained GDNF delivery via a CAC ECT platform was explored *in vitro* and *in vivo*. Also, the bioactivity and therapeutic efficacy of GDNF delivered from the gel were evaluated in dystrophic RCS rats.

## Materials and Methods

### Ethics Statement

All animal experiments were performed in strict accordance with the requirements of the Cap. 340 Animals (Control of Experiments) Ordinance and Regulations, and all relevant legislation and Codes of Practice in Hong Kong. All the experimental and animal handling procedures were approved by the Committee on the Use of Live Animals in Teaching and Research in The University of Hong Kong (CULATR #2529–11 and #3250–14). Rats were deeply anesthetized with xylazine (15mg/kg body weight) and ketamine (60mg/kg body weight). All efforts were made to minimize suffering. Topical analgesic 0.5% Alcaine (Alcon) was applied. Painful stimuli, including pedal withdrawal reflex and tail pinch, was applied to ensure the rats were under anesthesia before and during the operation. Breathing rate and heart rate were regularly checked to ensure that they were of adequate depth, rate and normal character (heart rate: 300–500 bpm and respiratory rate: 70–110 bpm). Warming devices such as heat mat and warm chamber were applied to maintain the body temperature of the rats after anesthesia. Minimal dissection with appropriate instruments, minimal blood loss and gentle handling of tissues were ensured during the surgery to minimize trauma. Prophylactic antibiotic ointment (Tobrex®, Alcon) was topically applied post-operation, and the rats were kept in a warm and humidified chamber to recover prior to transferring them back to their cages. Rats were checked daily for eye inflammation, evidence of weight-loss and signs of stress for the first 7 days after surgery or until the experimental endpoint, whichever occurs first. 15–20% weight loss of the rats was used as the humane endpoint in this project. If the rats experienced serious breathing problems or major blood loss during the intravitreal injection, they would be euthanized immediately by an overdose intraperitoneal injection of pentobarbital (Alfasan) at dosage of 100-150mg/kg to avoid unnecessary suffering.

### HEK293 Cell Culture

HEK293 cells over-expressing GDNF were kindly provided by Dr. P.T. Cheung, The University of Hong Kong [40]. Briefly, a 633bp wild-type (wt) murine GDNF cDNA was cloned into an expression plasmid vector pHM6 (Roche) encoding the GDNF short transcript. HEK293 cells were then transduced with this pHM6–633wtGDNF plasmid using Lipofectamine^TM^2000 Transfection Reagent (Life Technologies Inc.) at 4μg DNA/10μl. Cells were cultured in Dulbecco's modified Eagle's medium-high glucose (DMEM-HG, Life Technologies), supplemented with 10% fetal bovine serum (FBS, Thermo Fisher Scientific) and 1% penicillin/streptomycin (Life Technologies) at 37°C with 5% CO_2_. G418 sulfate (Merck) at 500μg/ml was supplemented for selection and maintenance of positive clones. Medium containing G418 was replaced every 3 days.

### Preparation of Collagen-Alginate Gel

ECT gels were manufactured based on Lee et al. [9]. Briefly, HEK293 cells were trypsinized using 0.25% Trypsin-EDTA (Gibco) and suspended in a composite solution consisting of neutralized rat-tail collagen solution type I at a final concentration of 2mg/ml and aqueous sodium alginate (Fluka, 70:30 ratio of guluronic:mannuronic acid) at a final concentration of 0.7% or 1% (w/v). In this study, the total cell number encapsulated per gel was 1x10^4^ or 2x10^5^ and the total gel volume was 2 or 4μl. These gels were about 10 and 20mm in length respectively. To manufacture cylindrical-shaped gels with uniform diameters, glass capillary molds (Sigma-Aldrich) were used. 2 or 4μl of the CAC-cell mixture was introduced into a 0.2M CaCl_2_ filled glass mold and incubated at 37°C and 5% CO_2_ for 1h for alginate and collagen gelation. These gels were immersed in a 0.2M CaCl_2_ bath for five minutes at room temperature and washed with 1x PBS before transferring to 6-well or 12-well culture plates (IWAKI) for further culture and evaluations.

### Cell Migration Assay

To study the cell immobilization power of ECT gels composed of different alginate concentrations, cell migration assay was carried out based on [9]. Briefly, the substrata of 12-well culture plates were coated with collagen gel at 0.5mg/ml to provide anchorage for the ECT gels and an enticement for the outward migration of HEK293 cells. 1x10^4^ cells were encapsulated in a 4μl composite gel with 0.7% or 1% (w/v) alginate. The ECT gel was embedded in the center of the semi-set collagen gel and was further incubated in 37°C for 20 minutes to allow the complete setting of the collagen slab. Culture medium was carefully added into the wells and replenished every 3 days. Morphology of ECT gels and encapsulated cells were studied with phase-contrast microscopy (n = 5). Experiments were performed in triplicates.

### Characterization of ECT Gel

2μl ECT gel with 1x10^4^ cells encapsulated in a CAC matrix of 1% alginate and 2mg/ml type I collagen was used for subsequent *in vitro* and *in vivo* characterizations. Conditioned media of ECT gel with or without cells was collected for GDNF analysis. The accumulated GDNF during the first week was collected at day 7 and the second week at day 14 (n = 3–5).

### Morphological Examination of ECT Gel

Temporal changes in the morphology of cultured ECT gels were examined by phase-contrast microscopy at day 7 and 14. Also, the microstructure of ECT gel at day 14 was studied by SEM (Hitachi S4800). ECT gels were fixed with 2.5% glutaraldehyde at 4°C overnight, rinsed and dehydrated in gradients of ethanol before critical point drying and gold sputtering for SEM analysis.

### Measurement of GDNF Secretion

The concentration of GDNF secreted from ECT gels *in vitro* and *in vivo* was evaluated by GDNF Emax® ImmunoAssay (Promega) at day 7 and 14 according to the manufacturer’s instructions. In this study, antibodies against human GDNF were used as human and murine GDNF exhibit over 90% amino acid sequence homology and are highly species cross-reactive [41]. Briefly, 96-well flat bottom ELISA plate (Thermo-fisher) was coated with anti-GDNF monoclonal antibody in carbonate coating buffer overnight at 4°C without shaking. Non-specific binding sites were blocked by 1X Block and Sample Buffer at room temperature for 1 hour before sample addition. After the addition of conditioned medium or diluted vitreous samples and serially diluted GDNF standard (0-1000pg/ml), the plate was incubated with shaking at 150rpm for 6 hours at room temperature (EDHSHO-1D, Wise). Then the plate was incubated with anti-human GDNF polyclonal antibody overnight at 4°C. Anti-Chicken IgY, horseradish peroxidase conjugate was added to the plate and incubated with shaking at room temperature for 2 hours. TMB One solution was added and the plate was incubated for 15 minutes without shaking at room temperature. Reaction was stopped upon the addition of 1N hydrochloric acid. Absorbance at 450nm was measured within 30 minutes with a multiplate reader (Elx800, BioTek). TBST wash buffer was applied in the washing steps.

The accumulative GDNF secretion from ECT gel with cells or without cells *in vitro* was compared at day 7 and 14. Conditioned medium were collected twice per week and were centrifuged at 5000rpm for 10 minutes (5424 centrifuge, eppendorf) to remove particulates and aliquoted for storage in -80°C before ELISA quantification. As for *in vivo* comparison, vitreous was extracted from healthy Sprague Dawley (SD) rats receiving injections of ECT gel with or without cells at post-natal day 28 (p28). These animals were sacrificed after 7 and 14 days of implantation.

### Cell Viability of ECT Gel

In order to evaluate the cell viability and morphology of implanted ECT gels, ECT gel explanted at day 7 was incubated with 2μM Calcein AM and 4μM Ethidium homodimer-1 (Molecular Probes) for 40 minutes at room temperature for the simultaneous staining of live and dead cells in Live/Dead assay. Results were evaluated by phase contrast and fluorescence microscopy.

### Animal Work

#### Animal models and surgical procedures

For *in vivo* studies involving the quantification of GDNF secreted, healthy male SD rats (Laboratory Animal Unit of The University of Hong Kong) were employed. As for evaluating the bioactivity of GDNF secreted from the implanted ECT gel, a well-established rat model with inherited photoreceptor degeneration, the pink-eyed dystrophic RCS/lav, was used. RCS/lav rats were obtained from Dr. Matthew LaVail (UCSF, UCSF School of Medicine) and were bred in the Laboratory Animal Unit of The University of Hong Kong (LAU). Male RCS/lav rats were used in this study. All rats were kept in a temperature-controlled room with 12-hour light/12-hour dark cycle and allowed free access to food and water. Operation was carried out at p28 for both types of rats.

Rats were anesthetized by intraperitoneal injection of a mixture of 15mg/kg xylazine and 60mg/kg ketamine (Alfasan lab). Analgesic topical 0.5% Alcaine (Alcon) was applied pre- and during operation. Under anesthesia, the pupil was dilated with topical 1% Mydriacyl (Alcon). The ocular surface and surrounding tissue were sterilized with diluted Betadine solution (Mundipharma A.G.), followed by cleansing with saline using a clean cotton swab. A 30G needle was used to introduce 2 holes in the cornea for the release of intraocular pressure during intravitreal injection. To visualize the intraocular space and retina during surgery, a circular piece of glass cover slip (10mm in diameter) was gently placed on the cornea with the support of a C-shaped corneal ring between the eye and glass slip. Saline was used to fill the space in between the glass slip, C-ring and the eye surface to allow better stability and adhesion of the slip on the corneal surface. After introducing a small incision on the conjunctiva at the superior temporal limbal region with a pair of conjunctival scissors to expose the sclera, an incision into the vitreous was made using a 20-gauge blade (Alcon) without disturbing the posterior capsule, lens and retina. ECT gel was injected into the posterior vitreous through a sterilized pipette tip with a fine opening (Sigma-Aldrich). The wound and conjunctiva were subsequently closed with 10–0 nylon suture. Topical antibiotic TOBREX ® (Alcon Laboratories Inc.) was applied to the eye for 2–3 days after the operation. At designated time points, rats were sacrificed by intraperitoneal injection of pentobarbital for vitreous GDNF concentration determination and retinal morphological examination.

#### *In vivo* GDNF delivery

**Fig 1:**
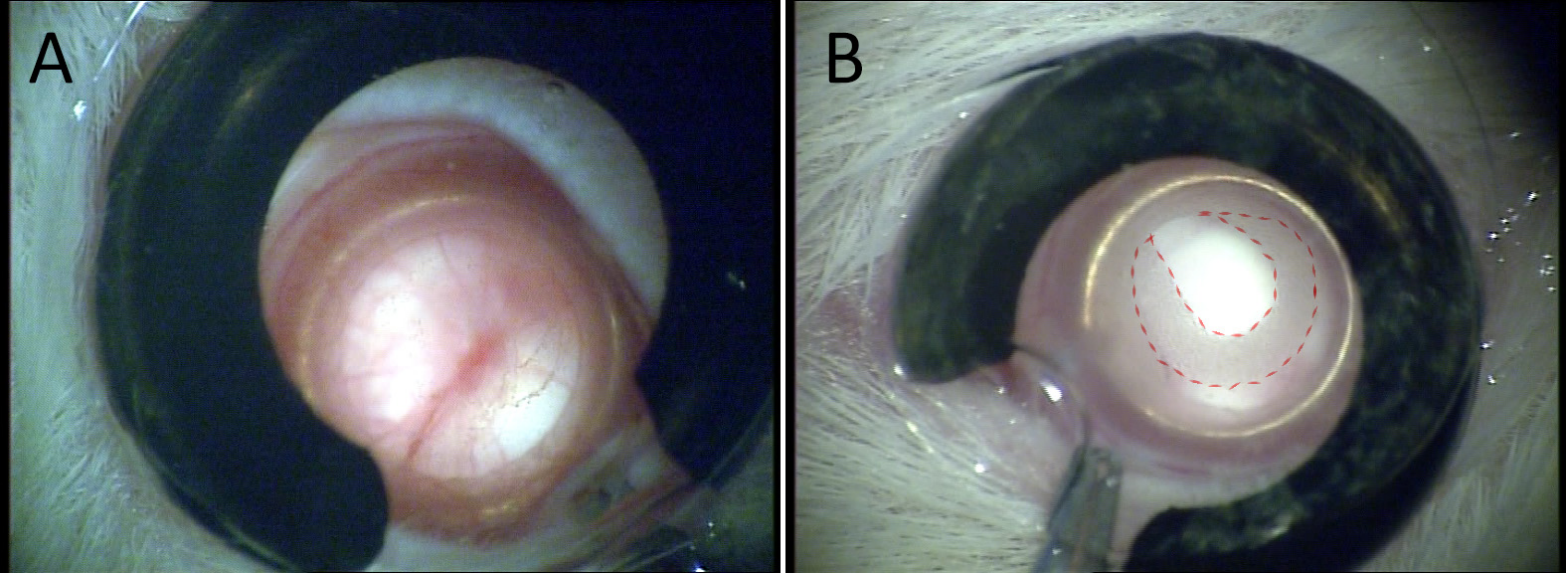
**Morphology of CAC ECT gel (A) without cell encapsulation and (B) with cell encapsulation (red dotted line) inside rat eyes under a surgical microscope.** The gel with no encapsulated cells was transparent and therefore could barely be seen. Photos were taken 1 week post-operation.

Nine rats were randomly divided into 2 groups: (1) ECT with 1x10^4^ cells/gel, and (2) ECT without cells (Fig 1). After 14 days of implantation, the animals were sacrificed for GDNF analysis. Vitreous was collected from the enucleated eyecups and filtered by centrifuging at 16000rpm for 15 minutes at 4°C (5415R centrifuge, Eppendorf) using an ultracentrifugation unit (Millipore). Samples were stored in -80°C before ELISA quantification. The cell-encapsulating ECT gel was collected at day 7 for morphological study and Live/Dead assay.

#### Bioactivity of GDNF delivered by ECT gel

As a pilot study to investigate the stability and therapeutic efficacy of ECT gels, two RCS/lav rats were implanted with gel containing 1x10^4^ cells. Retinal morphology at post-operation day 28 was compared with non-operated dystrophic rats. In a formal *in vivo* study to investigate the efficacy of the cell-containing ECT gel, 26 dystrophic rats were randomly divided into four treatment groups: 1) ECT with 2x10^5^ cells/gel (n = 9), 2) ECT without cells (n = 8), 3) surgical sham control (n = 3), and 4) unoperated group (n = 6). After 28 days of implantation, the animals were sacrificed for retinal morphological examination.

#### Retinal morphological examination

To allow effective penetration of fixative without damaging the retinal structure, eyecups were immediately fixed in 4% paraformaldehyde (PFA) in PBS for 1 hour after removing the cornea. After removing the lens, the eyecup was further immersed in PFA with regular agitation at room temperature overnight before paraffin embedding. Sagittal sections of 5μm parallel to the optic nerve were collected using a microtome (HM 315 Microtome, Microm). Hematoxylin and eosin (H&E)-stained retinal sections were examined and imaged with an upright microscope (Eclipse 80i, Nikon, Japan). The whole retina was imaged with about 15 microscopic fields at 400x magnification. These images were merged with Adobe® Photoshop®. Six regions of 810 pixels x 810 pixels were selected along the retina for ONL cell counting. They were: 1) superior peripheral, 2) superior mid-central, 3) superior central, 4) inferior central, 5) inferior mid-central, and 6) inferior peripheral. ONL cell count was quantified to evaluate the degree of the photoreceptor survival. The counts were normalized against their respective retinal length in the selection.

### Statistical Analysis

All data were expressed as mean ± SD. A blinded approach was used in all the experimental procedures. Verification of normality and homogeneity of variances were conducted before using parametric analysis. Non-parametric two-way ANOVA Friedman’s test was used to investigate the difference in accumulative GDNF concentration between ECT with cells and without cells over culture time. Mann-Whitney U test was used to compare the difference in accumulative GDNF concentration in vitreous between the treatment groups, ECT with cells and without cells. General linear model with repeated measures and appropriate post-hoc tests were applied to investigate the difference in ONL count among different treatments received and retinal locations. All statistical analyses were performed using SPSS 16.0. The significance level was set at 0.05.

## Results

### Alginate Concentration and Cell Immobilization

**Fig 2:**
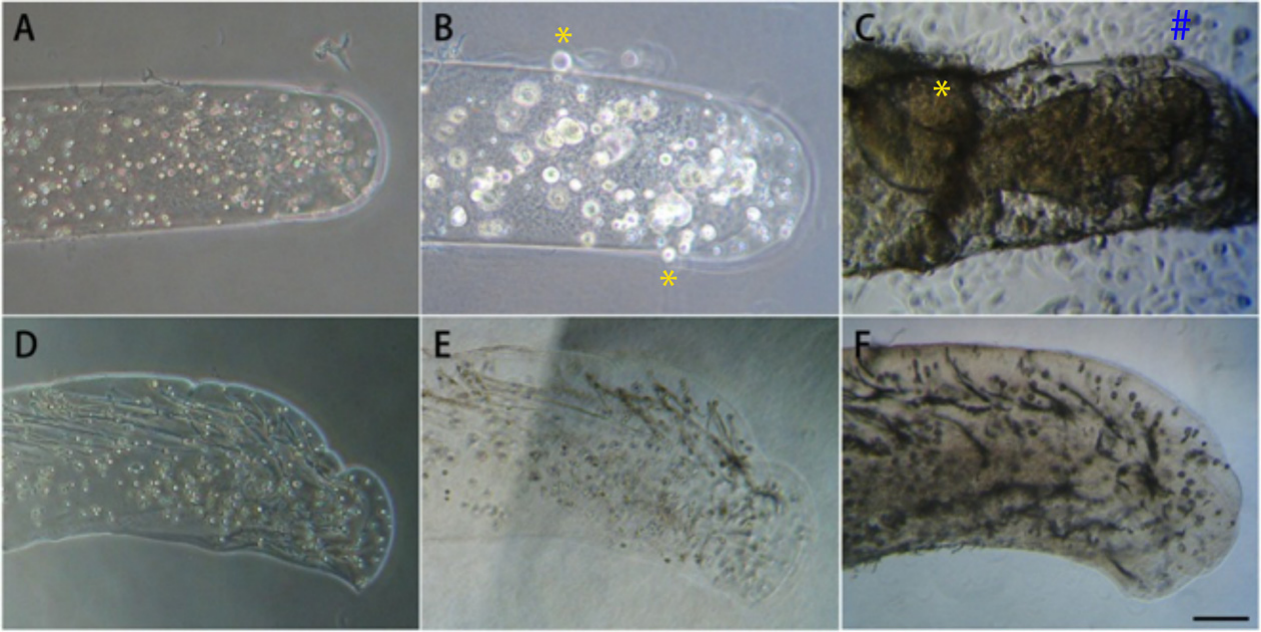
Temporal changes in cell immobilization at different alginate concentrations evaluated by cell migration assay. 1x10^4^ cell were encapsulated in a CAC matrix of (A-C) 0.7% or (D-E) 1% alginate and examined at day 0 (day of manufacturing), 7 and 28 (left to right). (A, D) The initial cell distribution was similar. (B) At day 7, cell colonies formation and mild migration of cells were observed in 0.7% alginate gels. (C) At day 28, colonies further expanded in size. Colonies located close to the outer regions of the gel or ones that had expanded to that region showed higher chances of cell migration from the gel to the surrounding (*, #). (D-F) Cell distribution in 1% alginate gel remained similar at day 0, 7 and 28. Scale Bar = 200μm.

To study the effect of alginate concentration on the cell immobilization properties of ECT gels, 1x10^4^ modified HEK293 cells with GDNF-expression were encapsulated in a 4μl CAC ECT gel with 0.7% (Fig 2A–2C) or 1% (Fig 2D–2F) alginate for cell migration assay over 28 days of culture. Cell distribution in both types of ECT gels was similar on the day of manufacturing (day 0) (Fig 2A and 2D). The distribution remained very similar over 28 days of culture for the 1% alginate group (Fig 2D–2F). As for the 0.7% group, cell colonies formation and mild migration of cells were observed at day 7. At day 28, these colonies further expanded in size. Colonies located close to the outer regions of the gel (Fig 2B and 2C) or ones that has expanded to that region showed higher chances of cell migration from the gel to the surrounding (Fig 2C). Also, colonies at the center regions of the gel fused together at day 28. 1% alginate was more effective in immobilizing cells than 0.7% alginate over 28 days of culture at this cell density.

### Characterization of ECT Gel

**Fig 3:**
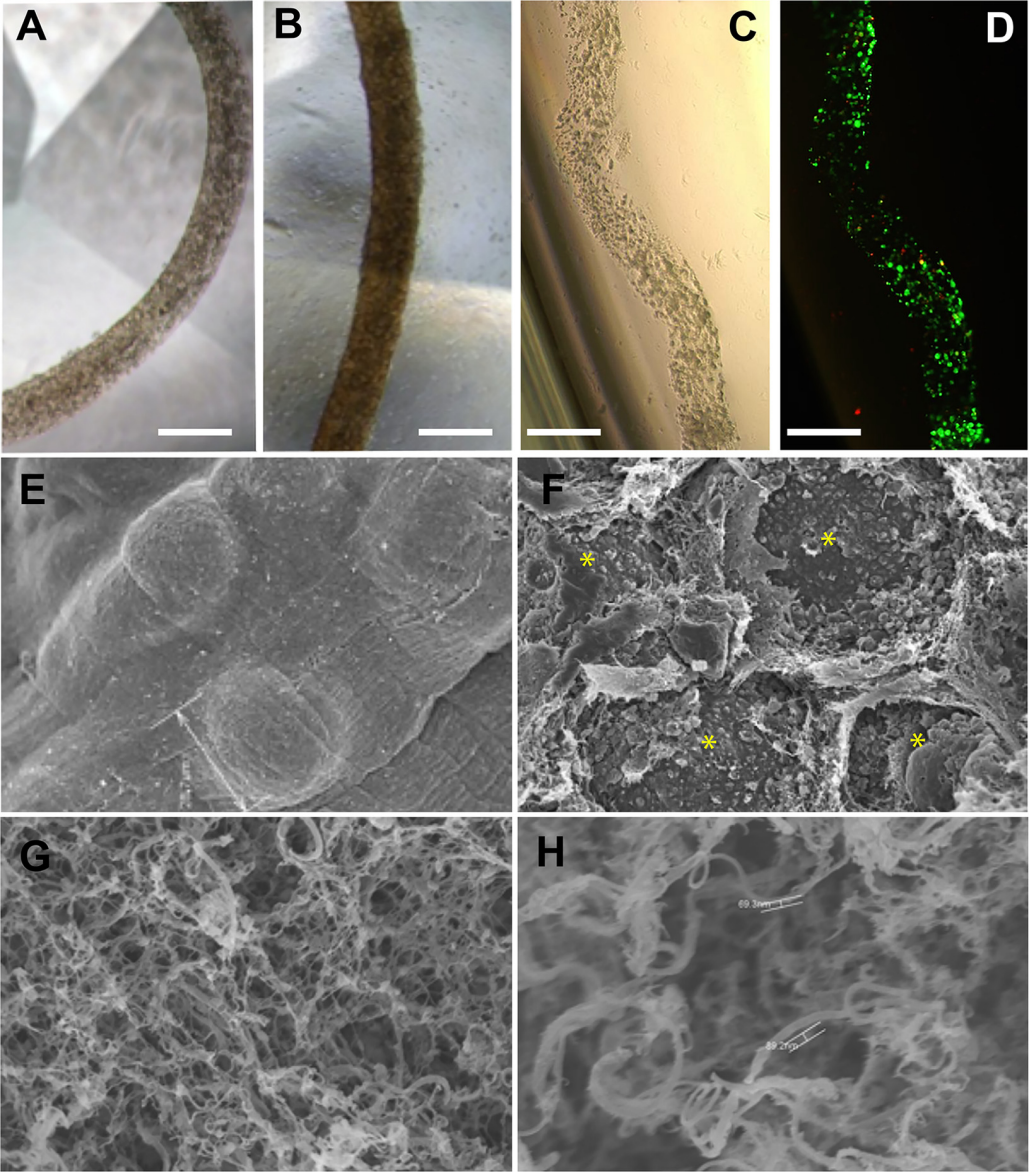
**Characterizations of 2μl CAC ECT gel with 1x10**^**4**^ **cells using (A-C) phase contrast imaging, (D) Live/Dead assay, and (E-H) SEM.** Morphology of ECT gel at (A) day 7 and (B) day 14 *in vitro*, and (C) explanted gel at post-implantation day 7 were evaluated by phase contrast microscopy. (D) Cell viability in the explanted gel was further studied by dual fluorescence staining of live (green) and dead (red) cells. Scale bar = 500μm. Microstructure of *in vitro* ECT gel at day 14 was examined by SEM: (E) gross appearance of the gel (1.4K x); (F) magnified view of the cross-section of the gel with cells entrapped in the composite gel matrix (2.2K x); (G) magnified view of the composite matrix with an IPN of collagen and alginate (25K x); and (H) magnified view of the hydrogel matrix showing a reconstituted collagen network (25K x). Since the ECT gel has a 3D structure, imaging under the same magnification may still result in varying apparent magnifications due to the difference in sample depth. For example, (H) was shallower than (G).

Doubled cell density by halving the total gel volume appeared to support better cell morphology at day 7 and 14 compared with lower cell density over 2 weeks of culture (Figs 3A, 3B and 1E). SEM imaging of the gel at day 14 showed entrapped cells within a meshwork-like CAC matrix (Fig 3E–3H). The CAC matrix was arranged in an IPN of alginate (Fig 3G) and reconstituted collagen (Fig 3H).

As a pilot study to investigate cell viability and gel morphology of the implanted ECT gel, the gel was retrieved from rat vitreous after 7 days of implantation (Fig 3C and 3D). The gross gel morphology and cell distribution after 7 days of implantation were similar to that of the pre-implanted gels., An increase in cell colony size was observed in both cultured and explanted gels at day 7 when compared to day 0. The colony size and density of the cells in the cultured gel (Fig 3A) appeared to be greater than that of the explanted gel (Fig 3C). Good cell viability was observed throughout the explanted gel with plenty of live cells and a very low amount of dead cells (Fig 3D).

### *In Vitro* and *In Vivo* GDNF Secretion

**Fig 4:**
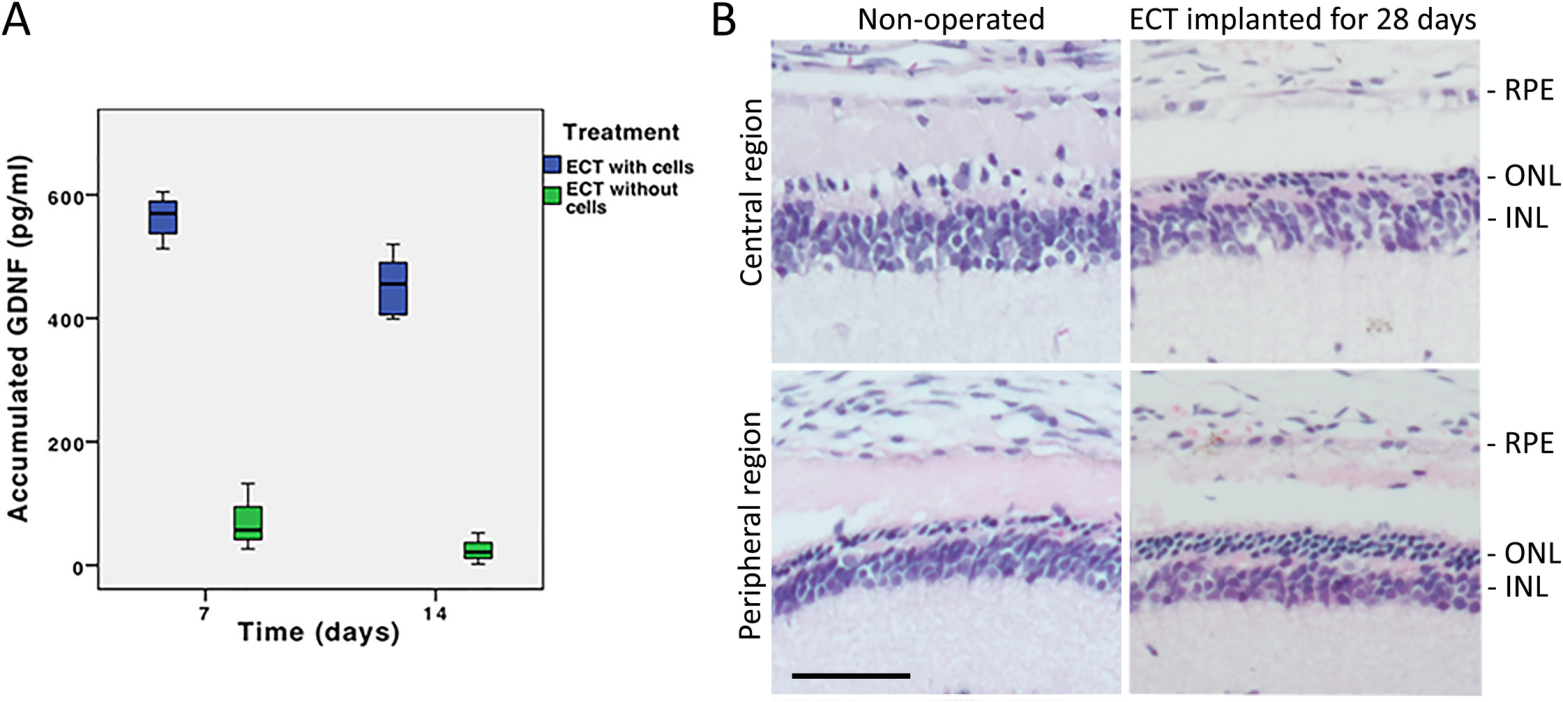
Concentration and bioactivity of GDNF delivered from the CAC ECT gel. (A) Box plot showing the accumulated concentration of GDNF secreted from the CAC ECT gel during the first and second week of culture. The ECT gel with cells showed a significantly higher GDNF release than the control group (p<0.045). (B) Photoreceptor survival after the implantation of ECT gel with 1x10^4^ cells (right) was compared to that of the non-treated control (left) at the central (top) and peripheral retinal regions (bottom). Better cell and spatial pattern retention in the ONL were detected after treatment with cell-containing ECT gel. Scale bar = 50μm.

Compared with the control ECT gel without cells, continuous GDNF delivery from the cell-encapsulating gel was detected in culture (Fig 4A) and rat vitreous for at least 14 days by GDNF ELISA. For *in vitro* accumulated GDNF level, non-parametric Friedman’s test showed a significant difference between the treatment groups (p<0.042). A slight decrease in the accumulated GDNF concentration was also detected in the second week compared to the first week. As for accumulated *in vivo* GDNF level, Mann-Whitney U test indicated a significant difference between the cell-encapsulating gel (1628.53±204.56 pg/ml) and the gel without cells (25.24±0.92 pg/ml) by day 14 (p = 0.016).

### Bioactivity of GDNF Delivered from ECT Gel

In a pilot study using ECT gel with a lower cell density, rats implanted with gel containing 1x10^4^ cells showed a better photoreceptor nuclei retention in the ONL than non-operated rats after 28 days of implantation at p56 (Fig 4B). For the peripheral retinal region (about 150μm from the peripheral end of retina), the ONL density of ECT-implanted rats and non-treated rats were 538±46 and 278±73 cells/mm respectively. Also, ONL in the treated rats was thicker than that in the non-treated rats. As for the central retinal region (about 150μm away from the optic nerve), ONL density of the ECT-implanted rats and non-treated rats were 271±36 and 128±34 cells/mm respectively.

**Fig 5:**
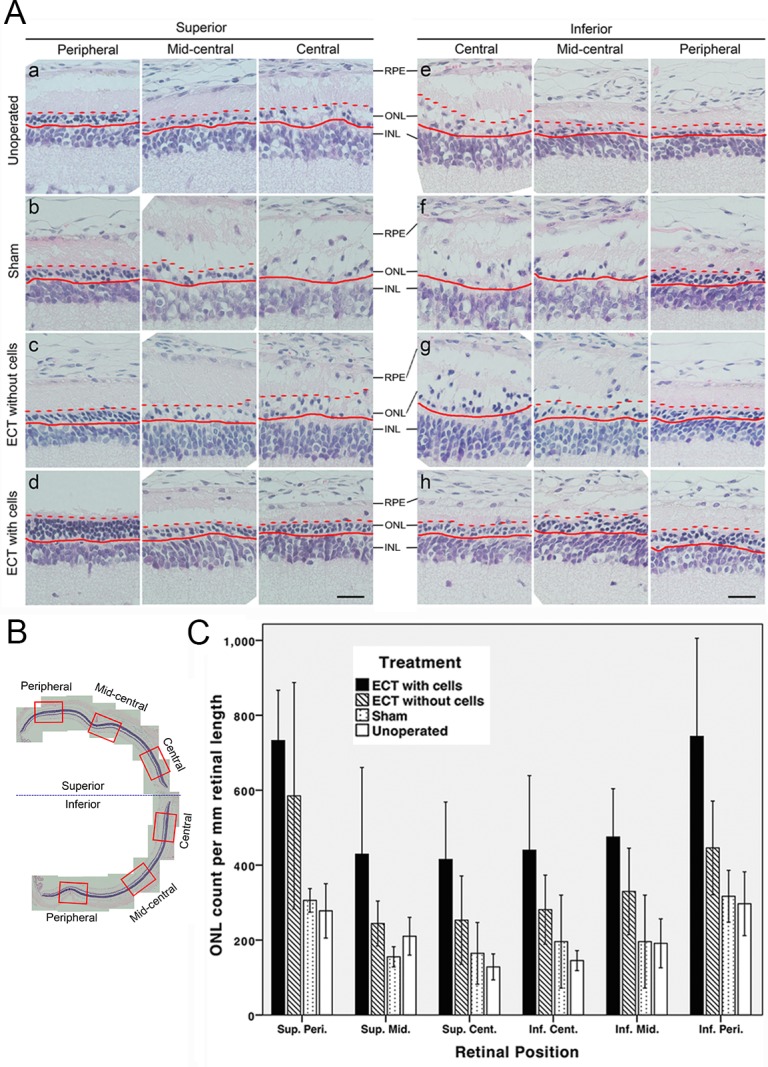
Photoreceptor survival in dystrophic RCS/lav rats evaluated by H&E staining. (A, B) Six retinal areas studied were: peripheral, mid-central and central regions of the (a-d) superior and (e-h) inferior retina respectively. (A) H&E staining at p56 showing the retinal morphology of rats receiving: (a, e) no operation, (b, f) surgical sham operation, (c, g) ECT gel without cells, and (d, h) ECT gel with 2x10^5^ cells for 28 days. The ECT with cells group showed better-aligned and thicker ONL than the other control groups. Preservation of photoreceptors at the periphery was better than that of the central areas in all treatment groups. Straight red line denoted the inner boundary of the ONL, adjacent to the inner nuclear layer (INL). Dashed red line represented the outer boundary of the ONL, adjacent to the RPE. Transiting from the peripheral to central retinal regions, ONL cells became more dispersed and the outer ONL boundary became unclear, especially in the (b, f) sham and (g) ECT without cells groups. Scale bar = 25μm. (C) ONL count was significantly affected by both treatments received (p<0.0005) and retinal location (p<0.0005). Among the treatments received, ONL count in the ECT with cell group was significantly higher than the other treatment groups, namely ECT without cells (p = 0.006), sham (p<0.0005) and non-operated group (p<0.0005). As for the retinal location, ONL count in the peripheral retina was higher than the central retina.

In a formal study on the efficacy of cell-containing ECT gels, rats implanted with gels containing 2x10^5^ cells (Fig 5A-d and 5A-h) showed more photoreceptors over all retinal regions of examination (Fig 5B) than other control groups, including ECT without cells (Fig 5A–c and 5A-g), surgical sham control (Fig 5A-b and 5A-f), and non-operated control (Fig 5A–a and 5A-e). H&E-staining revealed better ONL alignment and thickness in the rats receiving cell-encapsulating gel. General linear model with repeated measures showed that ONL count at p56 was significantly affected by both treatment received (p<0.0005) and retinal location (p<0.0005) (Fig 5C). For treatment received, Bonferroni post-hoc test showed that the ONL count in the ECT with cells group was significantly higher than the other treatment groups, including ECT without cells (p = 0.006), sham (p<0.0005) and non-operated groups (p<0.0005). In which, the ONL count in the ECT with cells group was at least one-fold higher than the sham and non-operated groups over all six areas of examination. As for retinal location, Bonferroni post-hoc test showed that the ONL count in central retinal regions was significantly lower than peripheral regions. The superior central has a significantly lower count than the superior peripheral (p = 0.002), and the inferior central was significantly lower than the inferior peripheral (p = 0.019). Additionally, cross comparisons between the central and peripheral regions of the superior and inferior retina showed similar results. The superior central ONL count was lower than the inferior peripheral (p = 0.04), whereas the inferior central has a lower count than the superior peripheral (p<0.0005).

## Discussion

We have previously reported that the incorporation of alginate in 3D collagen cultures of GDNF-secreting HEK293 in: (1) a composite CAC matrix form, or (2) a two-phase form with collagen ECT microspheres embedded in alginate, improved cell immobilization properties of the collagen ECT scaffolds, supported cell growth, and controlled the pattern of GDNF secretion [9]. In this study, the composite CAC ECT system was further developed into an injectable gel for sustained drug delivery in the posterior eye. In this study, the ECT gels were fabricated under physiologically relevant conditions, which involved mild temperature and ionic concentrations to initiate sol-gel formation, to allow simultaneous encapsulation of living cells. SEM revealed that the CAC matrix formed under the current protocol was arranged in an IPN with cells entrapped within a randomly arranged alginate and collagen matrix.

The survival and proliferation of mammalian cells are tightly regulated by their microenvironment through cell-extracellular matrix interactions and cell-cell adhesion. In a CAC system, alginate polymers presents no intrinsic cell-binding domains and minimal protein adsorption while type I collagen has peptide motifs for direct and indirect cell-binding. With minimal interaction in alginate, cells encapsulated in the 3D CAC matrix showed a reduced cell growth when compared to unconstrained growth in monolayer culture. Comparing with alginate gels of higher concentrations, i.e. 2 and 4%, Lee et al. reported better cell viability, and more importantly, much higher GDNF secretion rate in 1% gels [9]. Based on these findings, optimization of alginate concentrations at the lower end was carried out in this study. To determine the effectiveness of <1% alginate gels in cell immobilization, cell migration assay at 0.7% was also performed. The cell migration assay demonstrated that a higher alginate concentration was necessary for better cell proliferation control and prevention of cell leakage from the ECT gel. Similar to previous reports on proliferative cells encapsulated in CAC or alginate matrix, lower alginate concentration was associated with higher initial viability but faster spheroid formation in the ECT gel [9, 42]. Since cell leakage was already observable at day 7 for the 0.7% gel, this group was determined to be unfit for prolonged *in vivo* drug delivery applications. Hence, further quantification on its cell viability was not performed.

An increase in initial cell density showed better cell growth at 14 days of culture with cell colonies gradually increasing in size. When cells were encapsulated in a CAC or collagen matrix, they adhered and pulled on the collagen molecules, leading to the remodulation of their microenvironment. Such force was generated from cytoskeletal assembly and cell movements initiated by the formation of stable integrin-mediated attachments between collagen and cells. Depending on the concentration and degree of cross-linking of collagen in the CAC composite matrix, cells faced different levels of resistance to deform the matrix [43]. Higher density of cells might create a stronger pull for a tighter arrangement of collagen around them, and hence, through improving the cell-cell and cell-matrix interactions, cell growth and proliferation could be enhanced. Slower cell proliferation was previously reported when 3T3 fibroblasts were encapsulated in alginate gel at low seeding density when compared to intermediate density [42].

Continuous GNDF delivery from the ECT gel was detected in culture and in the eyes of healthy SD rats for at least 14 days. The gel was well-tolerated and no host tissue was detected on its surface. Also, there were no observable side effects or complications in rats due to CAC ECT implantation. No animals were rejected from this study based on the aforementioned humane endpoint criteria. GDNF was released at around 70 pg/ml/day in culture conditions. Due to a smaller distribution volume in SD rat eye, higher GDNF concentrations of 1.6 ng/ml were detected *in vivo* by day 14. It was higher than the normal amount of GDNF present in native SD rat retinal tissue, which was around 6.1 pg per μg total retinal protein [44]. After normalization with rat vitreous volume, GDNF secretion rate was around 140 pg/day. To gain a better understanding of the gel viability and morphology after implantation, the ECT gel was retrieved at day 7 for Live/Dead analysis. The explanted gel showed healthy cell colonies with a similar distribution as the pre-implanted gel. Colonies in the explanted gel were smaller in size compared with that of the cultured gel at day 7. Slower cell growth may be attributable to the more stringent ocular environment for the survival of transplanted cells when compared to *in vitro* cultures. The difference in oxygen and nutrient availability in the eye may also affect the rate of drug delivery. Clinical trials with the neurotrophic factor releasing ECT system, Renexus® (Neurotech), implanted in patients with retinal degeneration reported positive safety profile and ability to produce sustainable drug levels [45]. In a pharmacokinetic analysis on Renexus®, vitreous drug concentration of “high dose” implant was found to remain relatively stable at around 46-54pg/day. However, the *in vivo* secretion rate was about 10–15 folds lower than that from explants (600-2800pg/day). A similar fold difference was observed in a rabbit model [46]. Further studies on pharmacokinetics and cell viability in the explanted ECT gels after longer periods of implantation were warranted for the better understanding in the performance and stability of CAC-based platforms *in vivo*. Although the GDNF concentration in rat eyes has declined, it should not impact the drug delivery application significantly as photoreceptor survival was detectable in dystrophic rats after 28 days of ECT gel implantation. Since the majority of CAC gel characterization was performed at day 14 in our previous study [9], this time point was chosen to evaluate our initial design with 1x10^4^ cells. Based on our pilot data, where ECT with 1x10^4^ cells resulted in limited ONL cell retention effect, higher cell concentration of 2x10^5^ was applied in a similar design to test for neuroprotective effects in the subsequent formal morphological rescue study.

To evaluate the bioactivity of GDNF delivered, ECT gel was implanted in dystrophic RCS rats at p28. Rats receiving ECT with cells showed better ONL retention than control rats receiving ECT without cells, sham or no treatment. Retina morphological studies showed a similar degeneration pattern across all treatment groups. ONL cell loss in the central retinal was more severe than the peripheral regions. In the pilot study with ECT containing 1x10^4^ cells, ONL nuclei density and thickness of the treated rats were about 1 fold higher than the non-treated rats at the peripheral retinal region. As for the central retinal region, where photoreceptor degeneration was most severe, although the apparent ONL thickness was similar in both treatment groups, ONL nuclei density of the ECT-implanted rats was about one fold higher than that of the non-operated rats. Also, the spatial arrangement of ONL nuclei in the treated rats was better than that of the control group. The rats implanted with gel containing a higher initial cell number (2x10^5^) appeared to show better photoreceptor survival in yielding better ONL organization and higher ONL count (733±134 vs. 538±46 cells/mm retina with 1x10^4^ cells/gel at peripheral retina, and 415±153 vs. 271±36 cells/mm retina with 1x10^4^ cells/gel at central retina). When compared with the sham and non-operated controls, ONL count in the rats receiving ECT with 2x10^5^ cells/gel was at least one fold higher across the retina. Also, although no significant alterations were observed in the retinal morphology, more investigations would be needed to evaluate the temporal changes in retinal response to the gel on cellular and molecular levels. Such an absence in acute response may be contributed by the immunoprivileged status of the eye.

As the gelation process of Ca^2+^ and alginate is inherently reversible, Ca-alginate scaffolds may slowly dissociate through the gradual exchange of gelling divalent Ca^2+^ ions with sodium ions, or in the presence of calcium chelators or non-gelling cations upon long-term intravitreal implantation. Alginate hydrogel stability and long-term encapsulation power can be improved by forming stable cross-links with bifunctional crosslinkers or polymers such as poly-l-lysine, poly-l-ornithine, chitosan, and poly (ethylene glycol) [47–52].

## Conclusions

In order to circumvent repeated invasive intravitreal injections and improve drug availability at the neural retina, an implantable ECT gel with a composite collagen-alginate hydrogel matrix was developed to provide continuous GDNF delivery in the vitreous for treating posterior eye diseases. Alginate concentration and initial cell density were important factors to be considered when optimizing ECT systems with proliferative cells. Gels with lower alginate concentration yielded higher initial cell viability but faster spheroid formation while increasing initial cell density encouraged cell growth. The gel was able to support and control cell growth, and achieved sustainable GDNF delivery for up to 14 days *in vitro* and *in vivo*. Also, gels implanted in dystrophic Royal College of Surgeons rat eyes for 28 days retained photoreceptors while those containing higher initial cell number yielded better photoreceptor survival. In summary, the current ECT gel has shown to be a feasible solution to delivery GNDF into the vitreous for treating degenerative retinopathies. This study contributes towards the development of CAC hydrogel-based ECT implants for sustained-release of future therapeutics for treating posterior eye diseases.
